# Advancing the Discovery of Emerging Contaminants: A Leap in Technology and Data

**DOI:** 10.1021/acs.est.6c03027

**Published:** 2026-05-26

**Authors:** Wenhui Qiu, Pablo Gago-Ferrero, Juliane Hollender, Frederic D. L. Leusch, Susan D. Richardson, Saer Samanipour, Huahong Shi, Zhanyun Wang

**Affiliations:** † State Key Laboratory of Soil Pollution Control and Safety, Guangdong-Hong Kong Joint Laboratory for Soil and Groundwater Pollution Control, School of Environmental Science and Engineering, 255310 Southern University of Science and Technology, Shenzhen 518055, China; ‡ 203229 Institute of Environmental Assessment and Water Research, IDAEA-CSIC, 08034 Barcelona, Spain; § Swiss Federal Institute of Aquatic Science and Technology, Eawag, 8600 Dübendorf, Switzerland; ∥ Institute of Biogeochemistry and Pollutant Dynamics, 27219 ETH Zürich, 8092 Zürich, Switzerland; ⊥ Australian Rivers Institute, School of Environment and Science, Griffith University, Southport, Gold Coast, Queensland 4222, Australia; # Department of Chemistry & Biochemistry, 2629 University of South Carolina, 631 Sumter St., Columbia, South Carolina 29208, United States; ∇ Van ’t Hoff Institute for Molecular Sciences (HIMS), University of Amsterdam, Amsterdam 1090, GD, The Netherlands; ○ Queensland Alliance for Environmental Health Sciences (QAEHS), The University of Queensland, 20 Cornwall Street, Woolloongabba, Queensland 4102, Australia; ◆ State Key Laboratory of Estuarine and Coastal Research, 12655 East China Normal University, Shanghai 200241, China; ¶ Aquatic Resources Research Institute, Chulalongkorn University, Bangkok 10330, Thailand; †† Empa—Swiss Federal Laboratories for Materials Science and Technology, 9014 St. Gallen, Switzerland; ‡‡ National Centre of Competence in Research (NCCR) Catalysis, ETH Zürich, 8093 Zürich, Switzerland; §§ University of Southern Denmark, Department of Green Technology & Danish Institute for Advanced Study, 5230 Odense, Denmark

**Keywords:** emerging contaminants, chemical universe, analytical technology, artificial intelligence, future-oriented assessment framework, safe and sustainable by design

## Abstract

Emerging contaminants pose substantial threats to ecosystems and public health. While advances in analytical techniques and chemicals assessment have revealed the impacts of contaminants such as per- and polyfluoroalkyl substances, pharmaceutical residues, and microplastics, knowledge of the vast chemical universe remains limited. This Perspective examines the evolving attention toward emerging contaminants and drivers of their discovery. It identifies key challenges, including methodological limitations in detecting diverse chemical classes, fragmented data landscapes, and delays in translating scientific evidence into regulatory action. We highlight how technological and methodological innovations, particularly advanced analytical technologies, artificial intelligence, and future-oriented assessment frameworks, can shift paradigms in identifying and managing emerging contaminants from reactive to predictive. Critical to this transition is the adoption of open and FAIR principles (Findable, Accessible, Interoperable, and Reusable) and harmonized data standards to enable integration of heterogeneous evidence streams. Furthermore, we advocate coupling early warning systems with “Safe and Sustainable by Design” approaches to prevent regrettable substitution and reduce hazards at the source. Coordinated action across academia, regulators, industry, and funders is essential to establish proactive prevention as the norm in emerging contaminant management.

## Graphical abstract

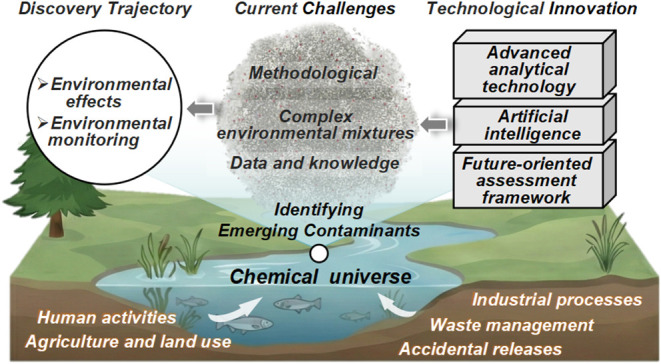

## 1 Introduction

Emerging contaminants (ECs) are chemical contaminants with increasing evidence and recognition as threats to ecosystems and/or public health, often also termed as “contaminants of emerging concern” (CEC). They are not necessarily new chemicals. Rather, many have been released and present in the environment for decades due to human activities.^1,2^ These contaminants are now widely detected in the environment and have garnered growing attention due to their potential risks.^3^ Despite growing knowledge on the environmental behavior and health risks of these contaminants, most have not been adequately managed under current regulations.

At the same time, the identification and management of emerging contaminants still face considerable challenges and delays. Many additional chemicals may be emerging contaminants, but are not yet captured by commonly used analytical methods^4,5^ or sufficiently addressed in existing research. For example, over 3,50,000 chemicals and mixtures have been registered in different parts of the world for production and use.^6^ In contrast, bibliometric studies show that only a limited number of chemicals (19,776 substances, less than 10%) have been analyzed in environmental media.^7^ When transformation products are also considered, the actual proportion of tracked chemicals is likely substantially lower, leaving the vast majority of potential emerging contaminants untracked. This limitation stems from current sampling, detection and identification methods, which are not effective in detecting and monitoring many chemical substances, including polymers and UVCBs (unknown or variable composition, complex reaction products, or biological materials).^8–10^

Existing studies show that emerging contaminants are pervasive across nearly all aspects of modern life, including consumer products,^11^ agricultural practices,^12^ and industrial processes.^13^ These factors highlight the urgent need for comprehensive and coordinated action to identify and address emerging contaminants, through enhanced vigilance, proactive research, and effective management, before they cause irreversible harm to ecosystems and public health.

Learning from past experiences, we summarize the discovery trajectory and key driving factors for uncovering emerging contaminants (Section 2) and outlines current challenges (Section 3) in this Perspective article. We further explore how technological and methodological advancements, particularly advanced analytical technologies, artificial intelligence (AI) and future-oriented assessment framework, have enhanced and can enhance the identification of emerging contaminants (Section 4). By enabling early warnings, these novel approaches can shift the paradigm in identifying and managing emerging contaminants from reactive to predictive.^14^ Building on these insights, we present several action-oriented recommendations to guide the future development of the emerging contaminant field, extending beyond identification toward solution-oriented approaches that safeguard both the environment and public health (Section 5).

## 2 Historical Lens: Learnings from the Past 60 Years

### 2.1 Discovery Trajectory of Emerging Contaminants

In the early days, the identification of certain emerging contaminants was often initiated by the observation of unexpected ecological or human health effects. For example, the discovery of polycyclic aromatic hydrocarbons (PAHs) can be traced back to 1775, when elevated cancer incidence was reported among chimney sweeps in the United Kingdom due to occupational exposure.^15^ Recognition of the environmental and health relevance of polychlorinated biphenyls (PCBs) began in the 1930s, when occupational exposure was linked to skin and liver damage in workers.^16^ Most recently, the observation of wild salmon mortality led to the identification of 6PPD-quinone, a previously unrecognized toxic transformation product of the tire rubber antioxidant 6PPD, as reported in a 2020 study using effect-directed analysis.^17^ Similarly, natural and synthetic hormones in wastewater have been identified as drivers of endocrine disruption in receiving waterways.^18^

In recent years, advances in analytical techniques have opened new possibilities by enabling the initial detection of certain substances in a wide range of environmental and biota samples for subsequent hazard and risk investigations. For example, disinfection byproducts (DBPs) were first identified in 1974 when Rook in The Netherlands and Bellar et al. in the United States independently detected trihalomethanes (e.g., chloroform) in chlorinated drinking water.^19,20^ Subsequent toxicological studies established their association with cancer risks, leading to the establishment of regulatory limits by the U.S. EPA in 1979.^21^ Similarly, per- and polyfluoroalkyl substances (PFAS), produced since the 1940s, began to attract widespread concern after several were widely detected in wildlife samples around the globe in the late 1990s,^22^ with subsequent research on their hazards and risks leading to calls for management.^23–25^ Likewise, the systematic identification of pharmaceutical residues emerged in the 1990s and early 2000s, driven by nationwide screening of drinking water contaminants in Germany and the United States and growing awareness of their endocrine-disrupting effects.^26–29^ Moreover, the identification of microplastics in the marine environment by Thompson et al. (2004) is considered to have renewed scientific interest in microplastics.^30,31^ Subsequent studies then demonstrated their bioavailability, toxicity, and potential associations with human disease risk, elevating them to a globally recognized class of emerging contaminants.^32^

As awareness of and societal demand for clean and healthy environment have intensified, identification of emerging contaminants has progressively shifted from serendipitous discovery toward more systematic and proactive approaches.^33,34^ This transition is primarily characterized by the application of complementary systematic strategies. These include systematic screening of chemicals on the market,^6,35^ using empirical testing and computational models, to evaluate hazardous properties (such as carcinogenicity, mutagenicity, endocrine disruption, reproductive toxicity, persistence, bioaccumulation potential). Such systematic screening enables proactive identification of potential emerging contaminants for environmental detection and further assessment. Additionally, systematic surveillance of air, water, soil, and biological samples is being developed to identify potential emerging contaminants, informing the testing and assessment of their ecological and health impacts.^36^ Together, this ongoing evolution toward proactive, systematic screening and surveillance has substantially enhanced the society’s capacity to early identify emerging contaminants, providing more effective pathways for pollution prevention and environmental governance.

To date, the chemical groups mentioned above, along with many others such as *ortho-*phthalates,^37^ bisphenols,^38^ and short-chain chlorinated paraffins,^39^ have progressively been identified and incorporated into regulatory frameworks. Collectively, this historical trajectory reflects a gradually deepening understanding of emerging contaminants, accompanied by advances in analytical capabilities (driving factor 1), expanded research on their impacts (driving factor 2), and increasing ability to collect, store and process large data sets (driving factor 3), as summarized in Figure 1.

**Figure 1:**
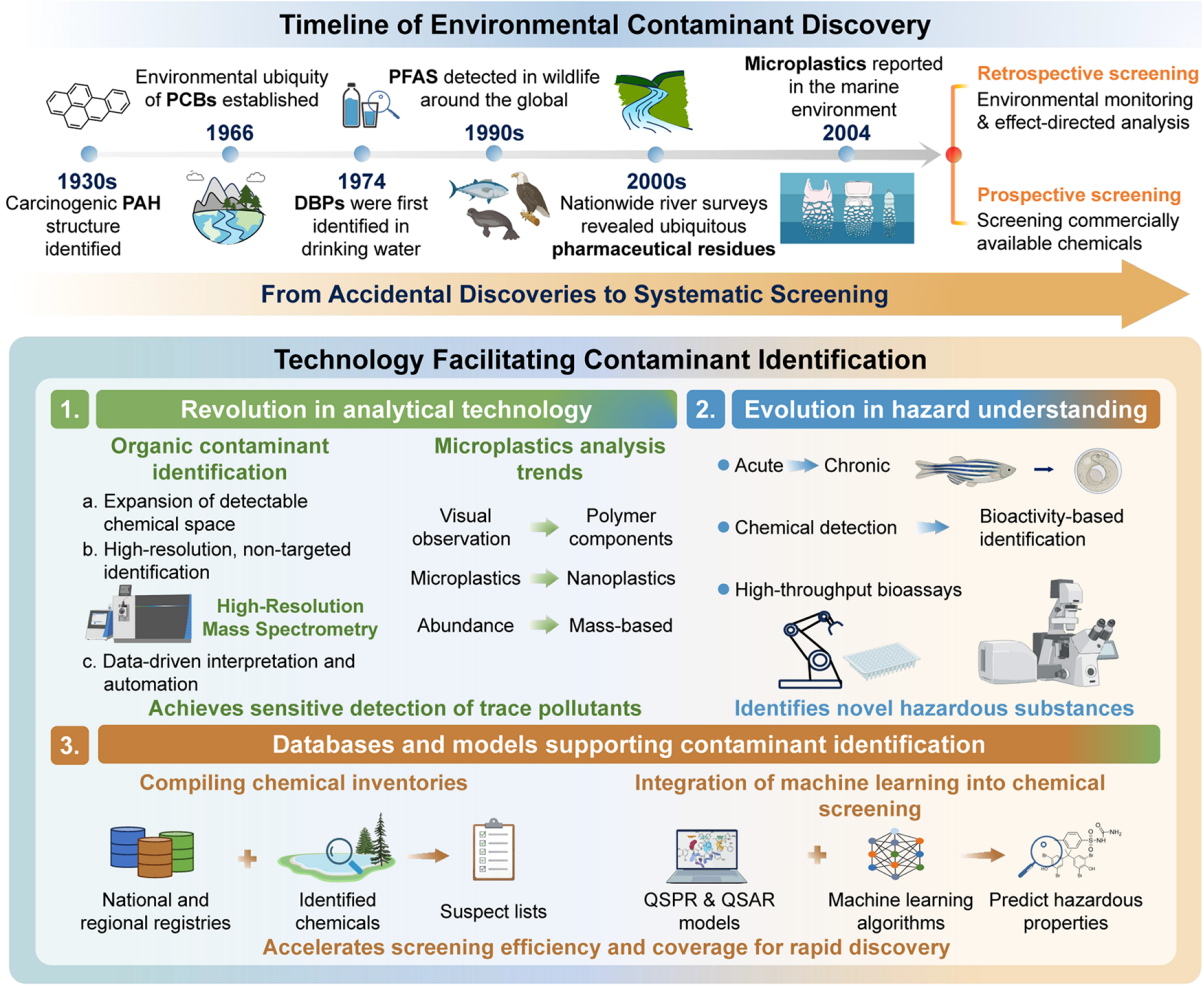
Temporal trends in the identification and understanding of emerging contaminants

### 2.2 Driving Factor 1: The Revolution in Analytical Technology

Although the term “emerging” appeared only in the 1990s, detection of known and unknown contaminants started much earlier. Mass spectrometry (MS) provided the selectivity and sensitivity to search for compounds outside the regulated chemicals in environmental samples. Since the 1970s, gas chromatography (GC)-MS with electron ionization and later chemical ionization has allowed the detection of volatile thermostable chemicals and, following derivatization, even more polar small molecules.^40^ Since the 1990s, liquid chromatography (LC) coupled to soft ionization such as mainly electrospray (ESI) and atmospheric pressure chemical ionization (APCI) has opened the window to more polar, thermally instable and larger molecules.

Various column materials (reverse phase, ion exchange, normal phase, hydrophilic interaction, mixed phases) in connection with polar and nonpolar solvents have been combined to cover a large chemical space.^4^ Tandem MS have enhanced the selectivity by coupling several MS with high-resolution mass spectrometry (HRMS), which provides more accurate (≤5 ppm mass deviation) and resolved (ratio of mass-to-mass difference ≥ 20,000) mass information data.^41^

Since 2005, time-of-flight (TOF) and Orbitrap instruments have been established in environmental analytics. By utilizing full scan mode with high mass resolution and high mass accuracy, these tools enable the detection of ionized molecules and provide fragment mass spectra for structure elucidation.

Similarly, with the continuous advancement of technology, the detection of microplastics in the environment has become more refined. The discovery of tiny plastics can be traced back to the 1970s,^42^ and the term “micro-plastics” was introduced in the literature in 1990.^43^ The identification of microplastics has evolved from initial microscopic observation to spectral discrimination of polymer components. Among these techniques, spectroscopy primarily relies on micro-Fourier-transform infrared spectroscopy (micro-FTIR) and micro-Raman spectroscopy.^44^ For the analysis of small-sized microplastics and even nanoplastics, advanced technologies and instruments such as surface-enhanced Raman spectroscopy (SERS) and nanoinfrared spectroscopy are required. However, the combination of spectral identification and microscopic observation only yields concentration data based on particle counts. It is required to use quantitative analysis methods such as pyrolysis-gas chromatography–mass spectrometry (Py-GC-MS) to obtain mass concentration.^45^ Future research on micro- and nanoplastics identification is trending toward the integration of imaging and spectroscopy techniques to achieve the simultaneous characterization of both particle number and mass concentrations.

### 2.3 Driving Factor 2: The Evolution of Understanding on Hazards, Including Toxicity

The discovery of emerging contaminants has been driven not only by improvements in analytical technology, but also evolving paradigms in chemicals assessment,^46^ which have expanded the scope of what is considered an emerging contaminant.

Initially, environmental toxicology focused primarily on acute toxicity end points, such as mortality and growth inhibition, often measured in whole organism bioassays. These end points, while ecologically relevant, were limited in scope and sensitivity. The identification of endocrine-disrupting chemicals in the early 1990s, together with the recognition that they can pose ecological threats even at trace concentrations, prompted the development of a range of new toxicity tests.^18^ This shift also redirected attention from acute and overt toxicity toward more subtle sublethal and chronic effects on endocrine, immune, developmental, and other biological pathways.^47^

Another major shift in chemicals assessment approach has been the recognition that mixtures of chemicals can produce “something from nothing”:^48^ many chemicals present at concentrations that individually produce no adverse effect combine to produce an adverse effect when present as a mixture. This has highlighted that a single-chemical risk assessment approach is deeply flawed, as emerging contaminants occur as complex mixtures in real-world environments. Instead, concepts such as concentration addition and independent action have enabled the development of new approaches to capture and quantify the cumulative biological activity of chemical contaminants in environmental samples, including effect-directed analysis and “iceberg modeling”.^49^ The latter is a computational method to determine what proportion of a bioassay-measured effect can be explained by chemically identified compounds, and what proportion remains attributable to unknown mixture.^49^ This has greatly expanded the understanding of the chemical universe, enabling the discover of “unknown unknowns”.

High-throughput bioassays have emerged as a powerful solution to these challenges. These assays, often based on cell culture or low complexity organisms, can rapidly screen large numbers of chemicals or environmental samples for specific modes of action, such as endocrine activity, oxidative stress induction, or genotoxicity. High-throughput bioassays can be used both for assessing the toxic properties of both old and new contaminants, as well as for quantifying the biological response in an environmental sample.^50^ This paradigm shift establishes the biological response itself becomes the metric of concern, rather than the mere presence or concentration of known contaminants. Consequently, this approach has been invaluable in identifying emerging contaminants that elude conventional chemical analysis, including transformation products, metabolites, and other unknown compounds.

High-throughput bioassays have also allowed the rapid development of the understanding of molecular toxicology; they enable the systematic testing of chemical activity across many different biological targets and the identification of modes of toxic action. This has greatly improved the understanding of mechanisms of toxicity, and to populate ever more complex Adverse Outcome Pathways (AOPs), which aim to link molecular-level perturbations to organism-level outcomes, providing a mechanistic bridge between in vitro data and ecological relevance.^51^

Together, these new methodologies are promising new tools to improve the understanding of risks posed by toxic chemicals in the environment,^52^ although scientists and regulators are still working out how to fully integrate them in 21st century risk assessment protocols.^52^ Ultimately, it is hopeful to cross the final frontier in ecological risk assessment that links comprehensive AOPs at multiple biological levels to predict adverse effects at the ecosystem level, incorporating biotic and abiotic modifying factors across multiple tropic levels.

### 2.4 Driving Factor 3: Support from Databases and Models

Advances in data storage, processing, and analysis have been another major driver of ongoing emerging contaminant identification. Such efforts typically begin with compiling chemical inventories that range in scope: from specific chemical classes^53,54^ to those used in specific applications or sectors,^55–57^ and to substances registered across jurisdictions.^6,58^ These inventories often include thousands to millions of chemicals, and are increasingly used as suspect lists in nontargeted analysis.^59^

The simultaneous evolution of computer power and algorithms has enabled to move from the identification of a few emerging contaminants using manual work to more high-throughput workflows.^60^ Such high-throughput workflows apply automatic peak-picking and deconvolution as well as isotope componentization to the thousands of signals, and then, assign molecular formula and structure applying rules and data science methods such as machine learning in connection with large chemical and spectra databases.^61^ For further candidate selection, physical-chemical properties to predict retention times and more recently chemical cross sections for ion mobility have been used, often in combination with meta data such as production volumes, exposure indexes, or biological and chemical transformation predictions as far as available.^62,63^

In addition, the large amount of molecular toxicology data produced by programs such as ToxCast enable computational modeling. Machine learning and quantitative structure–activity relationships (QSAR) models can be leveraged to learn patterns linking chemical structures to in vitro toxicity, greatly expanding the ability to predict risks associated with a growing universe of emerging contaminants.^64^ Early efforts employed quantitative structure–property relationship (QSPR) models to identify different types of potential emerging contaminants, including persistent (P), bioaccumulative (B), and/or toxic (T) chemicals, persistent organic pollutants (POPs), and chemicals of emerging Arctic concern.^58,65–67^ In recent years, *in silico* screening methods have become increasingly diversified through the integration of advanced machine learning algorithms. These tools facilitate high-throughput hazard identification directly from molecular graphs, fingerprints, and/or mass spectra,^68–73^ complementing existing hazard classification data reported by industry or regulatory agencies.^56,74–76^ Importantly, these studies significantly enhance the understanding of the scale and complexity of potential emerging contaminants in the global market. For example, they consistently suggest that the number of PBT or POP chemicals may far exceed those currently recognized under existing regulations, such as the Stockholm Convention.^58,77^ This highlights the critical need for sustained and intensified efforts to research, identify, and manage emerging contaminants effectively.

## 3 Current Status and Challenges in the Identification of Emerging Contaminants

Although technological advances have markedly accelerated the discovery of emerging contaminants, current approaches cannot yet comprehensively cover the vast chemical space and translate observed signals into actionable evidence in a timely manner, resulting in unnecessarily prolonged exposure to many potentially high-risk chemicals.

### 3.1 Methodological Challenges for Analytics

While HRMS-based nontargeted and suspect screening analysis primarily advance the front end of the workflow, namely detection and annotation of numerous features in a sample,^60,78^ a large gap remains in achieving comprehensive identification.^79,80^ This challenge is reflected in the confidence framework,^81^ under which only a small fraction—up to a few percent—of annotated features typically achieve Level 1 identification (confirmed structures), whereas most remain at Levels 2–3 or even Level 4 depending on the matrix, the MS2 coverage, the parameter settings, the number of in-house or purchased reference standards, and the effort invested, as demonstrated in a prenatal exposome and a groundwater study.^82,83^

Many features lack robust support from spectral libraries, isomers are difficult to resolve, and adduct formation, in-source fragmentation, matrix background, and inadequate instrument settings can lead to false-positive and false-negative identifications. Coverage is particularly limited for transformation products, metabolites, polymers, and UVCBs, which remain underrepresented in both databases and analytical workflows.^84,85^

Crucially, the absence of authentic reference standards and isotopically labeled internal standards often prevents high-confidence confirmation.^84,86^ In addition, any single sample-preparation and separation workflow typically accesses only a restricted portion of chemical space and cannot accommodate the full diversity of physicochemical properties.^87^

Consequently, the availability of standards and methodological capacity ultimately determines what fraction of signals can be elevated to confirmed with confidence levels, leaving a substantial share of environmentally relevant signals unresolved.

### 3.2 Challenges Associated with Complex Environmental Mixtures

#### 3.2.1 Disinfection Byproducts and Transformation Products

DBPs are particularly challenging because they are not manufactured like traditional chemicals, but are unintended consequence formed during water treatment. As the chemical structures of their precursors (mostly high-molecular-weight humic acid from natural organic matter) are heterogeneous and remain unknown, it is difficult to predict their structures. Obtaining standards to confirm their identification can also be challenging, requiring synthesis of new compounds.

To date, more than 1000 DBPs have been reported (with >700 structures confirmed),^88^ yet total organic halogen analysis indicates that ∼70% of halogenated DBPs in chlorinated drinking water is still unknown,^89,90^ with many of them being high molecular weight.^91^ This knowledge gap is concerning given that several human epidemiologic studies show increased bladder cancer, colorectal cancer, miscarriage, and birth defects,^88,89^ and many DBPs are cytotoxic, genotoxic, mutagenic, or carcinogenic, and are unregulated.^88,92–94^

Similar to DBPs, transformation products of anthropogenic contaminants can be challenging to uncover, despite knowing the precursors. This is because many transformation products are not always foreseeable due to many different abiotic and biotic pathways, different stability of the precursors and transformation products, and relevant local conditions. Some of the earliest environmental transformation products reported were from pesticides,^95^ followed by other contaminants, including pharmaceuticals, hormones, flame retardants, UV filters, PFAS, artificial sweeteners, benzotriazoles, algal toxins, and tire wear contaminants.^96,97^ In many cases, transformation products can retain the toxicity/activity of the original contaminants, or become more toxic.^98^

#### 3.2.2 Actual Environmental and Health Risks from Mixture Effects

Accurately assessing the true risks posed by environmental contaminants on human and ecosystems remains a formidable challenge for existing toxicity testing frameworks. Despite recent advancements, traditional paradigms often fail to capture the real-world risks posed by contaminants, which typically occur as complex mixtures. These mixtures, at trace concentrations, frequently exert sublethal effects (endocrine, reproductive, developmental, immune, and behavioral disruption) across sensitive life stages that remain poorly represented in conventional testing.

This is further complicated by micro- and nanoplastics, which act as complex carriers containing various additives, adsorbed pollutants, and inorganic particles. These particles are also subject to environmental processes such as aging, mineralization, and biofilm coverage, which can alter their surface morphology and chemical composition, potentially impacting their potency and environmental risk.^99^

### 3.3 Data and Knowledge Challenges

#### 3.3.1 Limited Coverage of a Vast Chemical Universe

As noted above, the chemicals currently studied represent only a small fraction of the chemical universe. Furthermore, research efforts are unevenly distributed across chemicals. Even among substances that have been investigated, studies tend to focus disproportionately on a limited number of well-known or legacy contaminants, leaving the majority of chemicals poorly characterized.^57,100^ This imbalance results in significant knowledge gaps and limits the representativeness of current chemical data to cover the large chemical universe, including for AI-driven property predictions.

This incomplete and uneven knowledge base also constrains current in silico approaches, constrained by both the applicability domain of current *in silico* methods and the extent of the knowledge of chemicals present in the global market. Most current *in silico* screening approaches rely on statistical models, including QSPR models as well as advanced machine- and deep-learning techniques. The applicability domain of these models is largely determined by their training sets. While different models vary in coverage, none are yet sufficient to encompass the full chemical universe represented in the global market.^58,101,102^ Continued efforts are needed to improve *in silico* screening of emerging contaminants. Key priorities include enhancing the collection of chemical identity information for substances on the global market and increasing the availability of empirical data, such as production volumes and hazardous properties, across a broader range of chemicals. Much of these data have already been generated by, or made available to, industry and competent authorities. Facilitating their public exchange through approaches that are FAIR (Findable, Accessible, Interoperable, Reusable) and accessible are critical and should be prioritized to maximize their utility.

Accordingly, substantial additional work is required to address the breadth and diversity of the global chemical inventory, particularly chemicals that are currently produced, widely used, or generated as transformation products in the environment. In particular, there is a critical need to move beyond well-known contaminants and expand research to include underrepresented and analytically challenging substance classes, such as polymers, UVCBs, organometallic compounds, and others.^8,9^ Broadening the research scope is critical for achieving a more comprehensive and realistic assessment of chemical risks in contemporary environmental systems.

#### 3.3.2 Challenges in Converting Data into Actionable Knowledge for Decision-Making

Emerging contaminant research is increasingly data-rich but knowledge-poor, with data generation is outpacing evidence integration, meaning more findings do not readily translate into faster or better action.^103^ One reason is that current evidence streams are diverse but often incompatible in data type, reliability, and interpretability. For example, nontargeted screening frequently yields only tentative annotations, with limited confirmation and little quantitative comparability.^86^ *In silico* outputs provide probabilistic predictions of chemical properties and characteristics (such as environmental fate and exposure), constrained by training and input data and applicability domains.^104^ Monitoring^105^ and epidemiological data sets^106^ are often sparse in space, time and chemical coverage, noisy, and heavily confounded. Also, these data are often scattered across the public domain. Therefore, significant additional time, expertise and resources are needed to collect, clean, and consistently integrate them to generate actionable conclusions.^107^

Bridging this gap to inform policy- and decision-making in a proactive and timely manner will require better data accessibility and comparability, including open data and libraries, harmonized data and uncertainty reporting, and standardized metadata. It will also require improvement of data integration, including decision-oriented knowledge engineering, and reproducible, auditable integration workflows across analytical, bioassay, and model outputs, for enhancing the regulatory transparency and acceptance of such evidence.

## 4 Technological Inflection Points: Tools That Are Expected to Shape Discoveries in the Future

### 4.1 Analytical Technologies

Mass spectrometric technologies have developed further in terms of sensitivity and selectivity (higher scan rate—i.e., more MS2 spectra, better mass accuracy, improved resolution), including new technologies such as the Astral detector,^108^ which narrows down the number of candidate structures for unknown signals. Still, mass spectra are not always informative enough to allow identification. Combination with other detection methods, currently used by only few groups, might become more widespread in the future. For example, nuclear magnetic resonance^109^ or infrared ion spectroscopy^110^ provides additional information for unambiguous structure elucidation, although sensitivity of the spectroscopic methods has still to be improved. Inductively coupled plasma mass spectrometry allows independent element identification, and thereby, help to narrow down candidates as demonstrated for PFAS.^111^

Coupling HRMS with various separation techniques such as supercritical fluid chromatography, reverse and normal phase, ion chromatography, or combinations has reduced the chemical space gap, recently, especially in the very polar field.^112,113^ At the same time, chemical and mass spectrometric databases^114,115^ as well as exposure-relevant suspect lists^59^ have increased, prediction of transformation products is enlarging,^116^ and *de novo* structure generation^117^ is becoming established so that the chemical space of candidates is increasing. Exploitation of millions of unannotated mass spectra in GNPS MassIVE with a neural network pretrained in a self-supervised way has provided a new way of structure annotations.^114^ Innovative data science methods are expected to facilitate emerging contaminant identification by integrating various analytical data (mass, isotope pattern, fragmentation spectra, retention time, intensity, ion mobility, ionization mode) and metadata (production volumes, consumption patterns, fate processes, metabolism, pathway predictions).

High-throughput analysis of hundreds of samples in the lab, as well as in situ analysis with mobile HRMS instruments,^118^ has expanded the spatial and time resolution. Together with automatic evaluation workflows, the time between detection to action can be reduced (early warning). This facilitates, for example, the elucidation of wastewater or surface water contaminants to stop their discharge or trigger the shut-down of nearby drinking water production in a timely manner. At the same time, the increasing number of measurement data enables successful application of statistical tools and AI to prioritize abnormalities over time or space, and subsequently identify emerging contaminants. An example is the daily monitoring of Rhine River combined with a time-trend analysis to prioritize, subsequently identify newly occurring intense signals, find their sources, and trigger actions.^60^ Portals such as the digital sampling freezing platform^119^ may store all these targeted and nontargeted data for river catchments, countries, regions, or even worldwide. By providing open access to these data alongside efficient data science tools, such platforms will be critical in accelerating the identification of relevant emerging contaminants.

The prioritization of HRMS features for identification based on hazardous properties is becoming more effective and widespread, a trend that is expected to continue. Using microfractionation in combination with downscaled bioassays and fast data processing workflows have allowed for high-throughput effect-directed analysis (HT-EDA) to identify toxicity-driving chemicals in samples.^120^ Similarly, high-throughput persistence or biodegradation testing, up to now mostly conducted on defined mixtures, can be effective for identifying persistent compounds or transformation products in environmental samples when combined with HRMS screening.^121^ Overall, the combination of various separation and detection technologies with increasing databases, predictions, and data science evaluation tools are expected to facilitate emerging contaminant identification in the future.

### 4.2 Transformative Role of Artificial Intelligence

Complementing empirical laboratory assays with *in silico* assessment reflects a fundamental paradigm shift in chemicals management.^122–125^ Machine learning and AI may serve as critical tools for identifying chemical risk signals by synthesizing heterogeneous data sets,^126^ from systematic reviews^127^ to industrial hazard mapping.^128^ Although still an emerging area of research, such approaches have already been applied to some extent, including predicting the occurrence of PFAS in groundwater and automating phenotype recognition for toxicity screening.^129,130^ Currently, the integration of Large Language Models (LLMs) and autonomous agents provides new ways to screen large amounts of text and extract essential information; for instance, applying human-like reasoning to unstructured accident reports represents a potential paradigm shift in early hazard discovery.^131–133^ Despite these advancements, “black-box” model opacity and need for manual validation of the results remain a significant hurdle for regulatory acceptance.^132^ Consequently, future efforts must prioritize transparency through Explainable AI as well as finetuning of LLMs for chemical-related text mining.^131^

Advancements in data fusion mark a critical shift toward integrated, AI-supported assessment frameworks. Bayesian networks (BNs) as well as Bayesian Neural Networks have shown to have the flexibility of fusing data from difference sources into networks with probabilistic dependencies being the main building block (i.e., the edges in the network). Additionally, these frameworks, while fusing different data sources, explicitly quantify uncertainty in environmental and chemical hazard assessment.^134^ Unlike traditional weight-of-evidence approaches, BNs enable dynamic updating as new data become available, improving the robustness and temporal stability of hazard classification.^135^ These hybrid network-based data fusion approaches have the advantage of reducing human bias, as new information is captured purely in a probabilistic manner.^136^ As a proof of concept, such hybrid modeling approaches have been employed for the fusion of historical concentration levels with potential sources, land use, and other environmental covariates to infer the concentration distribution of perfluorooctanoic acid (PFOA) in groundwater.^135,136^ Ongoing research increasingly emphasizes adaptive BN-machine learning frameworks to support real-time, multimodal decision-making in complex environmental and safety management contexts.

As the volume of experimental data increases and AI tools improve, models directly trained on multimodal data such as environmental DNA, persistence, and toxicity end points will enable the prioritization of chemicals associated with biodiversity loss or other negative ecological impacts. This approach will enhance the accuracy of emerging contaminant identification and facilitate early warning for these substances in the environment.

Overall, the ongoing advancement of AI concepts, algorithms, and tools is expected to enhance AI’s ability to assist in emerging contaminant identification and beyond. At the same time, AI tools remain dependent on high-quality input data, following the fundamental principle of “garbage in, garbage out”.^137^ Therefore, efforts to curate, identify, and annotate high-quality data sets are more important than ever and should be actively encouraged and supported.^126^

### 4.3 Future-Oriented Assessment Framework

The *One Health* framework provides a unifying lens to address emerging contaminants as cross-domain stressors affecting human health, ecosystems, and biodiversity simultaneously. Many emerging contaminants circulate across environmental compartments, food webs, and biological systems, ultimately resulting in chronic and cumulative human exposure through air, water, diet, and the built environment.^138^ Recognizing these interconnections shifts chemicals assessment from isolated end points toward systemic vulnerability and shared exposure pathways linking environmental change to human biological responses.^139^ Integrating exposomics, human biomonitoring, environmental monitoring, and effect-based tools within a *One Health* framework enables earlier detection of population-level risks and supports more targeted and preventive intervention strategies.^140^ This perspective is essential for anticipating long-term human health impacts in an increasingly complex and interconnected chemical environment.

Moving from *reactive regulation* to *proactive prevention* requires early warning platforms capable of detecting weak but meaningful signals of potential human and environmental risks before widespread impacts occur. Such platforms should integrate nontargeted HRMS screening, effect-based bioassays, exposure modeling, and AI-driven data mining and processing, chemical property prediction and pattern recognition across time and space. Rather than focusing on individual chemicals, early warning strategies should prioritize anomalies, emerging trends, and unexpected biological responses in environmental and human-relevant matrices.^141–143^ When supported by open data infrastructures and rapid analytical workflows, these platforms can inform timely decision-making, guide targeted follow-up investigations, and substantially reduce the lag between contaminant emergence, scientific recognition, and regulatory action.^144,145^

Together, these technologies are interrelated and mutually reinforcing, enhancing the identification and management of emerging contaminants and enabling more proactive, predictive, and integrated approaches to environmental and health risk management (Figure 2).

**Figure 2:**
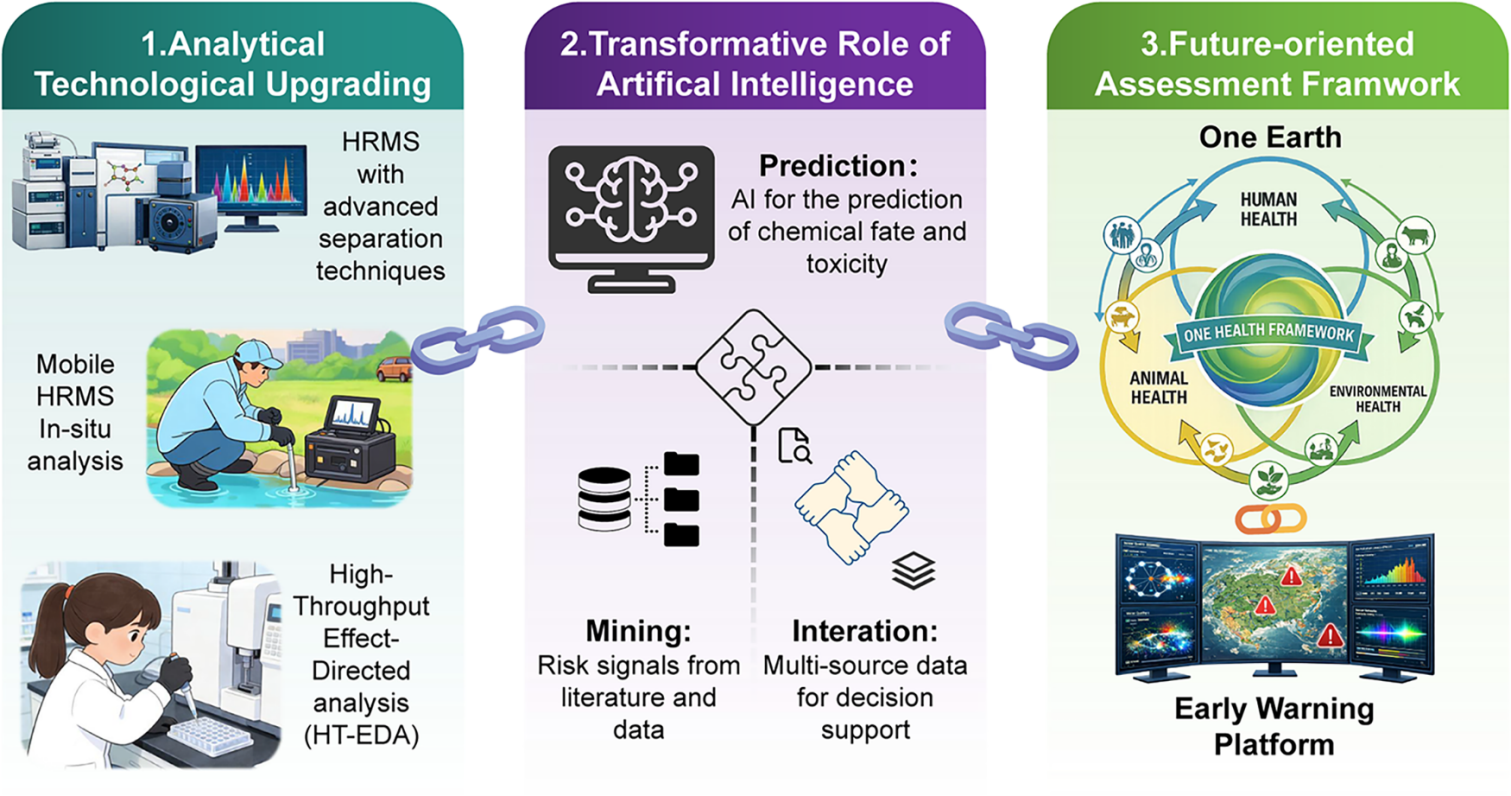
Triple technological revolution in emerging contaminant identification: analytical technological, artificial intelligence, and future-oriented assessment framework.

## 5 Call for Action

In the early warning, policy translation and governance of emerging contaminants, a main barrier is often not the lack of data, but the limited accessibility, interoperability and usability of data.^146^ Currently, nontargeted HRMS features, effect-based bioassay results, AI model predictions, monitoring, and epidemiological evidence are fragmented and typically inconsistent in terms of formats, metadata, quality control (QA/QC), and uncertainty reporting. Such methodological fragmentation hinders evidence integration, weakens cross-study comparability, and ultimately hampers the early capture and conversion of warning signals into actionable evidence.^146–149^ Therefore, scientists across sectors worldwide, funding agencies, journals, and data platforms should work together to promote a research ecosystem that fully aligns with the open and FAIR principles and addresses regulatory data requirements. We call for more open data practices and harmonized reporting standards,^4^ with priority given to critical requirements for reuse of data in crucial analysis of emerging contaminants that cover chemical identity, analytical parameters, detection and quantification limits, identification confidence, uncertainty characterization, and key contextual information. Additionally, shared terminologies and unique identifiers should be used to link occurrence data with use, emission scenarios, transformation pathways, environmental monitoring, biological toxicity, and exposure assessments, among others, to integrate fragmented data sets to actionable evidence.

To effectively address emerging contaminants, it is equally important to consider their potential replacements. Past experiences have repeatedly demonstrated the risk of regrettable substitution, where one emerging contaminant is replaced by another chemical that is later found to pose similar or other concerns, as exemplified by the substitution of PFOA with hexafluoropropylene oxide dimer acid (HFPO–DA), which shifted, rather than eliminated, concern within the PFAS family,^150^ or the replacement of bisphenol A with bisphenol S in plastics, which did not meaningfully reduce risk as both compounds pose comparable hazards.^151,152^

Several strategies can help mitigate this risk. One approach is to pursue nonchemical alternatives, such as advancing product and process design to eliminate the need for a given emerging contaminant altogether. Additionally, when substitution with another chemical is unavoidable, thorough assessments should be conducted to prevent regrettable outcomes. In this context, the European Union has introduced the concept of “Safe and Sustainable by Design”^153^ to guide the (re)­design of chemicals and materials toward safer and more sustainable solutions. Realizing this vision will require not only technical guidance, but also clear socio-economic incentives and regulatory drivers to promote industry adoption, including support for innovation and market uptake of safer alternatives.^154^ Currently, substantial efforts are underway to operationalize this concept,^155^ making it an important area to monitor closely.

We advocate an early warning paradigm in which meaningful anomalies in environmental and human-relevant matrices are detected promptly, triaged using effect-based and computational tools, to trigger and inform subsequent assessments, thereby reducing the lag between contaminant emergence and regulatory action.^156–158^ More importantly, early warning should be coupled to “Safe and Sustainable by Design” so that early signals and prioritization evidence can directly inform redesign and substitution decisions that reduce hazards and exposures at the source and avoid regrettable substitution.^159^ Achieving this transition will require coordinated action across academia, regulators, industry, funders, and journals so that proactive prevention becomes the norm in emerging contaminant management.
